# Factors associated with the perception of university social responsibility among dental students from two universities in the Peruvian capital: a multivariable regression analysis

**DOI:** 10.1038/s41598-024-74182-1

**Published:** 2024-10-07

**Authors:** Marysela Ladera-Castañeda, Elizabeth Paucar-Rodríguez, Jenny Cieza-Becerra, Miriam Castro-Rojas, José Escobedo-Dios, Ana Aliaga-Mariñas, Alberto Cornejo-Pinto, Luis Cervantes-Ganoza, César Cayo-Rojas

**Affiliations:** 1 Faculty of Dentistry and Postgraduate School, Research Group “Salud Pública – Salud Integral”, Universidad Nacional Federico Villarreal, Lima, Peru; 2 School of Stomatology, Universidad Privada San Juan Bautista, Lima, Peru; 3 Faculty of Stomatology, Universidad Inca Garcilaso de la Vega, Lima, Peru

**Keywords:** Perception, University social responsibility, Dental students, Sustainability, Dentistry, Health policy

## Abstract

Students play a key role in university social responsibility (USR), as they are the protagonists of change and the fundamental axis in the teaching-learning process. This study aims to assess the factors associated with the perception of university social responsibility among dental students from two universities in the Peruvian capital. This analytical cross-sectional study assessed 754 students from two Peruvian universities between May and July 2023. The study utilized a validated 51-item questionnaire to assess sociodemographic factors and dental students’ perception of USR. A Poisson regression model with robust variance and prevalence ratio (PR) was used to assess the influence of variables such as age, sex, marital status, type of university, academic year, and awareness of taking subjects related to USR. The significance was set at *p* < 0.05. The perception of USR was rated as poor, fair, and good by 16%, 67%, and 17% of the participants, respectively. According to the study, dental students from public universities were 68% more likely to perceive their USR education as poor than those from private universities (APR = 1.68, 95% CI: 1.06–2.66), and those who were aware of taking MSR-related courses were 37% less likely to perceive their USR education as poor (APR = 0.63, 95% CI: 0.40–0.98). Similarly, dental students attending public universities were 2.28 times more likely to perceive poor organizational management in USR compared to their counterparts at private universities (APR = 2.28, 95% CI: 1.51–3.44). Additionally, students in their first and second academic years were 62% and 57% less likely to perceive poor organizational management in USR compared to those in their sixth year (APR = 0.38, 95% CI: 0.19–0.79 and APR = 0.43, 95% CI: 0.20–0.95; respectively). Furthermore, students from public universities were 2.31 times more likely to perceive social participation in USR poorly compared to their counterparts from private universities (APR = 2.31, 95% CI: 1.63–3.26). Lastly, it was observed that students from public universities were 2.51 times more likely to have a poor overall perception of USR compared to students from private universities (APR = 2.51, 95% CI: 1.67–3.78). Acknowledging the diverse contexts and challenges inherent in public and private institutions, it can be concluded that more than half of the dental students had a fair perception of USR. Additionally, attending a public university was found to be a risk factor for having a poor perception of USR. However, taking a course related to USR and being in the early years of the program were identified as protective factors against a poor perception of organizational training and management in USR. It is recommended that students be involved in USR activities, including the Sustainable Development Goals, from the first years of their university education, especially in public universities.

**Supplementary Information:**

The online version contains supplementary material available at 10.1038/s41598-024-74182-1.

## Background

In recent years, universities have proposed reforms to respond to the challenges of globalization, sustainable development, technological progress, and knowledge. Various institutional strategies have been proposed to improve the interaction between universities and society and meet the needs of the population^1,2^. University social responsibility (USR) is a management policy that addresses the organizational and academic impact of universities^2–4^. This involves the collaboration of various university actors to promote ethical principles and social development, with the goal of training professionals who contribute to the sustainable growth of the nation^5,6^.

Universities have a crucial role in leading efforts to achieve the Sustainable Development Goals (SDGs) by 2030. They are recognized as important actors with broad convening capacity among different sectors of society. Therefore, university education is a fundamental pillar to achieve these goals^7,8^, and USR is the most appropriate management strategy to take on this challenge^5^.

In 2018, the Union of Latin American University Social Responsibility (URSULA) conducted a study involving 60 universities, 18 of which were Peruvian. The study reported that these Peruvian universities demonstrated strengths in cognitive issues. This was due to the generation of community projects, despite institutional licensing issues. However, these projects were not institutionalized^9^. On the other hand, these universities had little success in integrating the SDGs with community participation^5,9^, since many of them were still in the early stages of implementing USR despite having institutional licenses^5^.

Currently, many universities have integrated subjects related to USR into their curricula. These subjects include moral and civic education, which aim to train students in important skills such as decision-making, problem-solving, research, analysis, and negotiation. The goal is to form responsible professionals who are committed to solving social, economic, political, and environmental problems^10,11^. This situation should also be reflected in the dental field to promote the sustainability of oral health with equitable, ethical, high-quality, inclusive, and safe care, using resources effectively and efficiently^12^. Universities have a responsibility to instil the principles of social responsibility in future dentists during their training to respect and protect the care of current and future generations, minimizing the negative impacts of this profession^12^. However, research in health sciences has shown that many students lack knowledge and understanding of the implications of social responsibility^13,14^. Therefore, it is essential to investigate students’ perceptions of USR^15^, as well as universities’ commitment to social responsibility, and identify possible barriers and facilitators to its integration^11,16^.

Some studies in health sciences have reported that sociodemographic factors can influence students’ perception of USR. For instance, Bellido-Medina et al. (2019) found that 5th-year students had higher scores than 4th-year students. Additionally, female students had higher scores than male students^17^. Similarly, Mazud et al. found that younger students had a more positive perception than older students. Additionally, students in the preclinical area had a better perception of USR compared to those in the clinical area^18^.

This study aims to assess the factors associated with the perception of university social responsibility among dental students from two universities in the Peruvian capital. The null hypothesis stated that there are no factors associated with the perception of university social responsibility among dental students.

## Methods

### Study design

This cross-sectional, analytical, observational, and prospective study was conducted in students of the Faculty of Dentistry of the Universidad Nacional Federico Villarreal (UNFV) and the School of Stomatology of the Universidad Privada San Juan Bautista (UPSJB) from May to July 2023. The study was written following the Strengthening the Reporting of Observational Studies in Epidemiology (STROBE) guidelines^19^.

### Population and participant selection

Founded on 30 October 1963 in the city of Lima, Peru, the Universidad Nacional Federico Villarreal (UNFV) has 18 faculties and 60 professional schools, as well as a graduate school offering master’s and doctoral degrees, with a student population of approximately 22,670. In March 2020, the university received its institutional licence from the National Superintendence of Higher Education (SUNEDU), which guarantees the quality of its education. The Faculty of Dentistry, located in Lima, is an autonomous unit within the UNFV. At the time of the study, this faculty had two curriculum plans: the 2006 annual curriculum plan, which was in the process of being finalised, and the 2019 semester curriculum plan, which was in the process of being implemented^20,21^.

Founded in 1997, the Universidad Privada San Juan Bautista (UPSJB), located in Lima, Peru, with additional branches in San Borja, Chincha and Ica, has 4 faculties and 16 undergraduate schools, as well as a graduate school that offers second specialities and master’s degrees, with a student population of approximately 19,000. This university has been licensed by SUNEDU since November 2019. The Professional School of Stomatology, located in Lima, is part of the Faculty of Health Sciences. At the time of the survey, it was implementing its 2021 curriculum plan^22,23^.

**Fig. 1:**
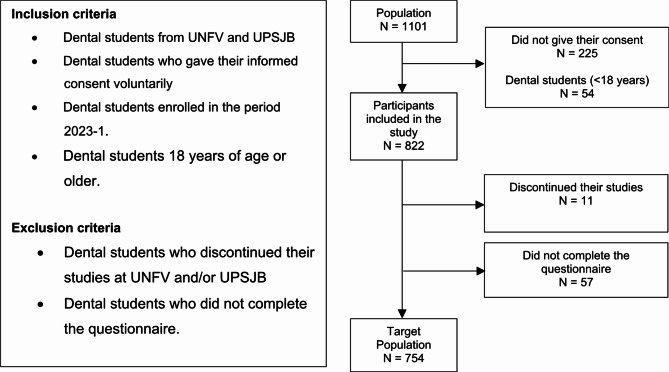
Participant selection flowchart.

The study population comprised 1101 dental students, with 397 from UNFV and 704 from UPSJB. The sample size was calculated using the Epidat 4.2 statistical package, with a confidence level of 95%, an expected proportion of 50% (to obtain the largest sample size), and an estimation error of 5%, based on a formula for estimating a proportion with a finite population. The entire study population was included, respecting the selection criteria, since the calculated sample size was *n* = 285. The total number of participants was *N* = 754, with 315 from UNFV (64 from the first year, 91 from the second year, 57 from the third year, 42 from the fifth year, and 61 from the sixth year) and 439 from UPSJB (72 from the first year, 72 from the second year, 107 from the third year, 86 from the fourth year, and 102 from the fifth year) [Fig. 1].

### Variables

The dependent variable was dental students’ perception of university social responsibility (USR), and the independent variables were age^17,24–26^, gender^17,24–26^, marital status^26^, type of university^27^, academic year^17,25,26^ and awareness of taking subjects related to USR^15^.

### Validation and application of the instrument

The study utilized a Spanish questionnaire named “University Social Responsibility Perception Survey” for students, which was adapted from Vallaeys et al.^28^. and previously used by Condori and Reyna^5^ and by ProCalidad of the National System of Evaluation, Accreditation, and Certification of Educational Quality (SINEACE)^29^. The questionnaire comprised 51 items, divided into two sections. The first section contained information on associated factors (age, sex, marital status, university of origin, academic year, and awareness of taking subjects related to USR). The second section consisted of questions on the perception of USR, which were distributed across three dimensions: D1 - perception about training in USR (items F1 - F14), D2 - perception about organizational management in USR (items G1 - G18), and D3 - perception about social participation in USR (items P1 - P13). The questionnaire consisted of Likert scale questions with answer values ranging from 1 to 5 (1: strongly disagree, 2: disagree, 3: neutral, 4: agree, and 5: strongly agree) [See supplementary material]. The content of the questionnaire was validated by three expert judges in the field of dental public health and research, who assessed its relevance, clarity, objectivity, and timeliness^30^. The questionnaire achieved an acceptable Aiken’s V score (V = 0.90, 95% CI: 0.86–0.93). To assess the instrument’s reliability, Cronbach’s α was calculated, resulting in a significantly acceptable value (α = 0.985; 95% CI: 0.981–0.989). In addition, very good internal consistency was observed for training (α = 0.961; 95% CI: 0.950–0.972), organizational management (α = 0.968; 95% CI: 0.958–0.976), and social participation (α = 0.965; 95% CI: 0.954–0.975). Additionally, 30 participants (15 randomly selected from each institution) were evaluated to assess the concordance of scores obtained at two different times within a 10-day period. The order of the questions was altered to avoid memory bias^31,32^. An acceptable intraclass correlation coefficient (ICC) was obtained (ICC = 1.00; 95% CI: 0.999–1.000; *p* < 0.001). The ICC test decision was based on the observed normal distribution, as determined by the Kolmogorov-Smirnov test for both the test and retest (*p* = 0.097 and *p* = 0.102, respectively).

To estimate the levels according to the overall score and according to their dimensions, Stanones’ rule [average (x̄) ± 0.75 (standard deviation)] was used to obtain the overall score for perception of USR: poor (≤ 147 points), fair (148 to 198 points), and good (≥ 199 points). For the USR training dimension: poor (≤ 46 points), fair (47 to 63 points), and good (≥ 64 points); for the USR organizational management dimension: poor (≤ 57 points), fair (58 to 79 points), and good (≥ 80 points); and finally, for the USR social participation dimension: poor (≤ 41 points), fair (42 to 56 points), and good (≥ 57 points). For purposes of performing the multivariate analysis, the levels of general perception and its three dimensions were dichotomized as Poor, and Regular/Good. Therefore, each cut-off point was validated according to Livingston’s K^2^ coefficient, obtaining a value of 0.990 for general perception and 0.977, 0.980 and 0.978 for its three dimensions, respectively; these being acceptable values.

### Procedure

The principal investigator administered the questionnaire to each student at the end of the lessons. This included the informed consent form explaining the purpose of the study and the associated risks and benefits. If students agreed to participate, they were directed to the questionnaire. The students filled in the questionnaire anonymously, as no personal details such as name, address or telephone number were requested. The study was conducted between May and July 2023. The principal investigator collected and tabulated the data using Microsoft Excel 2019. All researchers had access to the information, which was stored on a portable digital device with a password to maintain confidentiality. In addition, the results were emailed to students who requested it from the principal investigator. At the conclusion of the study, all data were destroyed for security reasons.

### Statistical analysis

Data analysis was performed using the SPSS v.28.0 statistical package. Descriptive statistics were calculated using relative and absolute frequency, mean, and median. Multivariate analysis was conducted using the prevalence ratio adjusted under the Poisson regression model with robust variance. A significance level of 5% (*p* < 0.05) was used for all tests.

### Bioethical considerations

The study adhered to the bioethical principles outlined in the Declaration of Helsinki^33^, specifically those related to confidentiality, freedom, respect, and non-maleficence. Approval was granted by the ethics committee of the Universidad Nacional Federico Villarreal with opinion number 01-2023-COMITE-DE-ETICA, dated 24 March 2023. Additionally, participants provided voluntary informed consent on the first page of the questionnaire.

## Results

The study found that the mean age of dental students was 22.5 ± 4.9 years, with 53.7% of the total being younger than 22 years. Women represented 66.0% of the participants, and 95% of the students were single. In terms of university type, 58.2% studied at a private university. The highest frequency of students was in their second (21.6%) and third academic year (21.8%). Finally, 50.1% of the students were aware that they were taking subjects related to USR [Table 1].

**Table 1:** Characterization of sociodemographic variables of dental students.

Variable	Category	Frequency	Percentage
Age group	< 22 years	405	53.7
	≥ 22 years	349	46.3
Sex	Female	498	66.0
	Male	256	34.0
Marital status	Single	716	95.0
	Married or cohabiting	38	5.0
Type of university	Public	315	41.8
	Private	439	58.2
Academic year	1st year	136	18.0
	2nd year	163	21.6
	3rd year	164	21.8
	4th year	86	11.4
	5th year	144	19.1
	6th year	61	8.1
Awareness of taking subjects related to USR	Yes	378	50.1
	No	376	49.9
Age	Mean	Median	SD
	22.5	21.0	4.9

SD: Standard deviation; USR: University Social Responsibility.

**Fig. 2:**
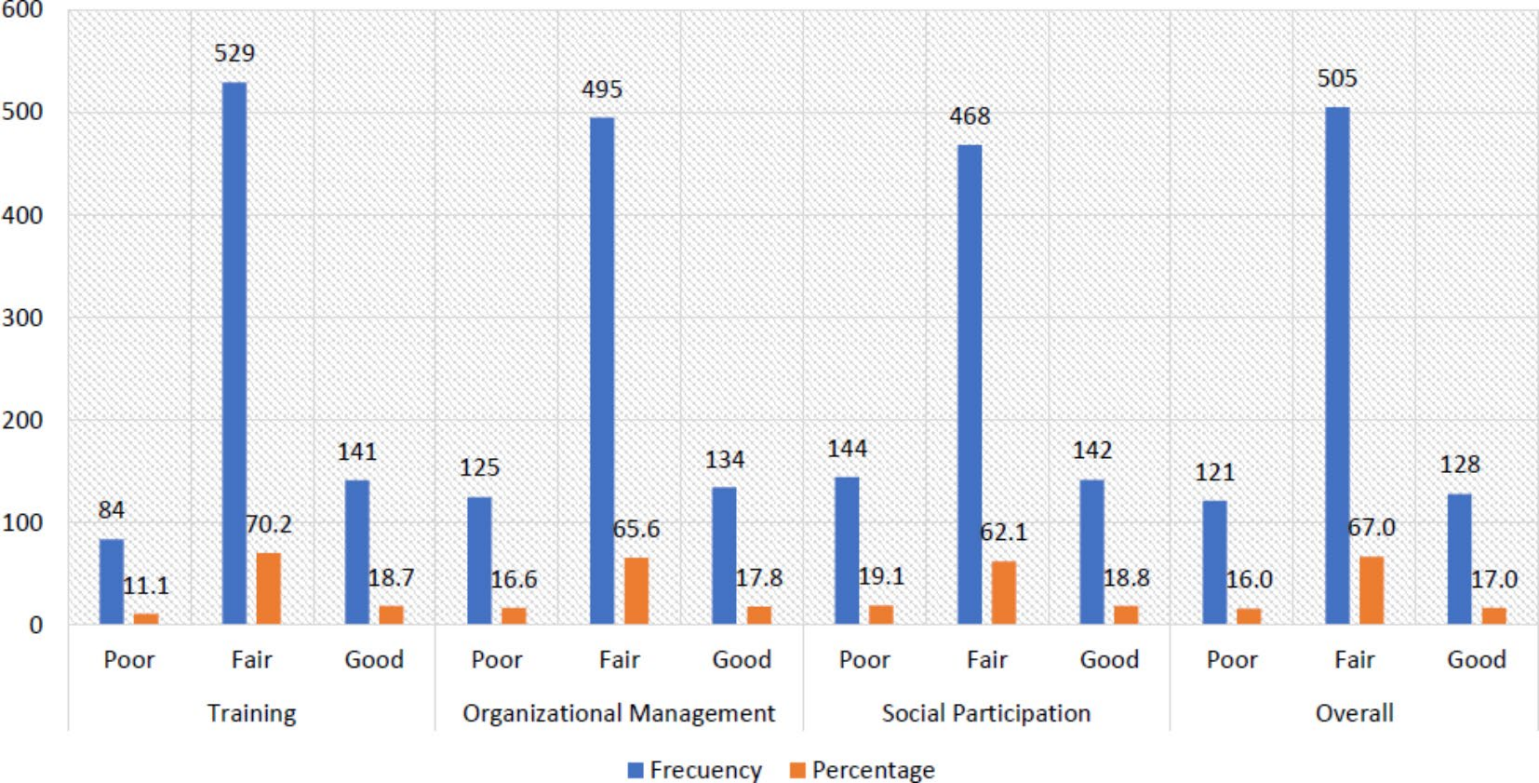
Frequency of dental students’ perception levels about USR overall and according to the dimensions of training, organizational management and social participation.

The study found that the frequency of perception of USR training was poor in 11.1% (95% CI: 8.9 − 13.4%), fair in 70.2% (95% CI: 66.9 − 73.4%), and good in 18.7% (95% CI: 15.9 − 21.5%). The perception of organizational management of USR was poor in 16.6% (95% CI: 13.9 − 19.2%), fair in 65.6% (95% CI: 62.3 − 69.0%) and good in 17.8% (95% CI: 15.0 − 20.5%). The perception of social participation in USR was rated as poor by 19.1% (95% CI: 16.3 − 21.9%), fair by 62.1% (95% CI: 58.6 − 65.5%), and good by 18.8% (95% CI: 16.0 − 21.6%). Finally, the overall perception of USR was rated as poor by 16% (95% CI: 13.4–18.7%), fair by 67% (95% CI: 63.6 − 70.3%), and good by 17% (95% CI: 14.3 − 19.7%) [Fig. 2].

The dependent variable in the crude model of the Poisson regression analysis with robust variance using Prevalence Ratio (PR) was the perception of USR training (Poor = 1 and Fair/Good = 0). Possible associated variables were age group, gender, marital status, type of university, academic year, and awareness of taking subjects related to USR. After adjusting the prevalence ratio (APR) of the model, it was discovered that dental students from public universities were 68% more likely to have a poor perception of USR training compared to those from private universities (APR = 1.68, 95% CI: 1.06–2.66). Additionally, students who were aware of taking subjects related to USR were 37% less likely to have a poor perception of USR training compared to those who were not aware of it (APR = 0.63, 95% CI: 0.40–0.98) [Table 2].

**Table 2:** Factors associated with dental students’ perception of USR training.

Variable	Category	Crude model					Adjusted model				
		β	PR	95% CI		p*	β	APR	95% CI		p**
				LL	UL				LL	UL	
Age group	< 22 years	-0.39	0.68	0.45	1.02	0.061	-0.15	0.86	0.50	1.48	0.583
	≥ 22 years		*Ref.*					*Ref.*			
Sex	Female	-0.28	0.76	0.50	1.14	0.180	-0.32	0.73	0.48	1.09	0.123
	Male		*Ref.*					*Ref.*			
Marital status	Single	-0.54	0.58	0.29	1.18	0.133	-0.56	0.57	0.28	1.16	0.123
	Married or cohabiting		*Ref.*					*Ref.*			
Type of university	Public	0.28	1.33	0.89	1.99	0.166	0.52	1.68	1.06	2.66	0.027**
	Private		*Ref.*					*Ref.*			
Academic year	1st year	-0.40	0.67	0.29	1.56	0.356	-0.10	0.90	0.34	2.39	0.837
	2nd year	-0.42	0.65	0.29	1.48	0.310	0.07	1.07	0.39	2.97	0.894
	3rd year	-0.07	0.93	0.43	2.00	0.852	0.41	1.51	0.64	3.58	0.352
	4th year	-0.34	0.71	0.28	1.79	0.466	0.37	1.45	0.49	4.30	0.501
	5th year	0.15	1.16	0.55	2.47	0.691	0.69	1.98	0.84	4.69	0.119
	6th year		*Ref.*					*Ref.*			
Awareness of taking subjects related to USR	Yes	-0.34	0.71	0.47	1.07	0.102	-0.47	0.63	0.40	0.98	0.039**
	No		*Ref.*					*Ref.*			
Model constant							-1.70	0.18	0.06	0.54	0.002

*Simple Poisson regression model (*p < 0.05, significant association); **Adjusted multiple regression model (**p < 0.05, significant association); PR: Prevalence ratio; APR: Adjusted Prevalence Ratio under Poisson regression model with robust variance; β: Coefficient of determination; 95% CI: 95% Confidence Interval; LL: Lower Limit; UL: Upper Limit. The adjustment variables were age group, sex and marital status.

The dependent variable in the crude model of the Poisson regression analysis with robust variance using Prevalence Ratio (PR) was the perception of organizational management in USR (Poor = 1 and Fair/Good = 0). Possible associated variables were age group, gender, marital status, type of university, academic year, and awareness of taking subjects related to USR. After adjusting the prevalence ratio (APR) of the model, it was discovered that dental students from public universities were 2.28 times more likely to have a poor perception of organizational management in USR compared to those from private universities (APR = 2.28, 95% CI: 1.51–3.44). Additionally, students in their first and second academic years were 62% and 57% less likely, respectively, to have a poor perception of organizational management in USR compared to students in their sixth academic year (APR = 0.38, 95% CI: 0.19–0.79 and APR = 0.43, 95% CI: 0.20–0.95; respectively) [Table 3].

**Table 3:** Factors associated with dental students’ perception of organizational management in USR.

Variable	Category	Crude model					Adjusted model				
		β	PR	95% CI		p*	β	APR	95% CI		p**
				LL	UL				LL	UL	
Age group	< 22 years	-0.76	0.47	0.33	0.66	< 0.001*	-0.28	0.76	0.46	1.25	0.277
	≥ 22 years		*Ref.*					*Ref.*			
Sex	Female	-0.06	0.95	0.68	1.32	0.747	-0.18	0.83	0.60	1.15	0.268
	Male		*Ref.*					*Ref.*			
Marital Status	Single	-0.11	0.89	0.45	1.78	0.752	-0.19	0.82	0.40	1.70	0.600
	Married or cohabiting		*Ref.*					*Ref.*			
Type of university	Public	0.70	2.02	1.46	2.80	< 0.001*	0.82	2.28	1.51	3.44	< 0.001**
	Private		*Ref.*					*Ref.*			
Academic year	1st year	-1.49	0.22	0.12	0.41	< 0.001*	-0.96	0.38	0.19	0.79	0.009**
	2nd year	-1.47	0.23	0.13	0.40	< 0.001*	-0.84	0.43	0.20	0.95	0.038**
	3rd year	-1.07	0.34	0.21	0.55	< 0.001*	-0.40	0.67	0.38	1.17	0.161
	4th year	-0.96	0.38	0.22	0.67	0.001*	0.05	1.05	0.50	2.21	0.899
	5th year	-0.65	0.52	0.34	0.80	0.002*	0.00	1.00	0.61	1.66	0.990
	6th year		*Ref.*					*Ref.*			
Awareness of taking subjects related to USR	Yes	-0.21	0.81	0.58	1.11	0.193	-0.26	0.77	0.54	1.10	0.152
	No		*Ref.*					*Ref.*			
Model constant							-1.30	0.27	0.12	0.64	0.003

*Simple Poisson regression model (*p < 0.05, significant association); **Adjusted multiple regression model (**p < 0.05, significant association); PR: Prevalence ratio; APR: Adjusted Prevalence Ratio under Poisson regression model with robust variance; β: Coefficient of determination; 95% CI: 95% Confidence Interval; LL: Lower Limit; UL: Upper Limit. The adjustment variables were age group, sex and marital status.

The dependent variable in the crude model of the Poisson regression analysis with robust variance using Prevalence Ratio (PR) was the perception of social participation in USR (Poor = 1 and Fair/Good = 0). Possible associated variables were age group, gender, marital status, type of university, academic year, and awareness of taking subjects related to USR. After adjusting the prevalence ratio (APR) of the model, it was discovered that dental students from public universities were 2.31 times more likely to have a poor perception of social participation in USR compared to those from private universities (APR = 2.31, 95% CI: 1.63–3.26) [Table 4].

**Table 4:** Factors associated with dental students’ perception of social participation in USR.

Variable	Category	Crude model					Adjusted model				
		β	PR	95% CI		p*	β	APR	95% CI		p**
				LL	UL				LL	UL	
Age group	< 22 years	-0.60	0.55	0.41	0.74	< 0.001*	-0.28	0.75	0.50	1.14	0.177
	≥ 22 years		*Ref.*					*Ref.*			
Sex	Female	0.12	1.13	0.82	1.56	0.449	0.05	1.05	0.77	1.43	0.772
	Male		*Ref.*					*Ref.*			
Marital status	Single	-0.23	0.80	0.44	1.44	0.449	-0.29	0.75	0.41	1.36	0.347
	Married or cohabiting		*Ref.*					*Ref.*			
Type of university	Public	0.61	1.84	1.37	2.48	< 0.001*	0.84	2.31	1.63	3.26	< 0.001**
	Private		*Ref.*					*Ref.*			
Academic year	1st year	-0.91	0.40	0.23	0.71	0.002*	-0.32	0.72	0.37	1.40	0.338
	2nd year	-1.15	0.32	0.18	0.57	< 0.001*	-0.49	0.61	0.30	1.27	0.185
	3rd year	-0.40	0.67	0.42	1.06	0.088	0.31	1.36	0.80	2.31	0.254
	4th year	-0.63	0.53	0.30	0.95	0.034*	0.43	1.54	0.75	3.16	0.238
	5th year	-0.22	0.80	0.51	1.26	0.346	0.46	1.58	0.95	2.61	0.077
	6th year		*Ref.*					*Ref.*			
Awareness of taking subjects related to USR	Yes	-0.14	0.87	0.64	1.16	0.337	-0.26	0.77	0.56	1.06	0.113
	No		*Ref.*					*Ref.*			
Model constant							-1.67	0.19	0.09	0.40	< 0.001

*Simple Poisson regression model (*p < 0.05, significant association); **Adjusted multiple regression model (**p < 0.05, significant association); PR: Prevalence ratio; APR: Adjusted Prevalence Ratio under Poisson regression model with robust variance; β: Coefficient of determination; 95% CI: 95% Confidence Interval; LL: Lower Limit; UL: Upper Limit. The adjustment variables were age group, sex and marital status.

The dependent variable in the crude model of the Poisson regression analysis with robust variance using Prevalence Ratio (PR) was the overall perception in USR (Poor = 1 and Fair/Good = 0). Possible associated variables were age group, gender, marital status, type of university, academic year, and awareness of taking subjects related to USR. After adjusting the prevalence ratio (APR) of the model, it was discovered that dental students from public universities were 2.51 times more likely to have a poor overall perception in USR compared to those from private universities (APR = 2.31, 95% CI: 1.63–3.26) [Table 5].

**Table 5:** Factors associated with dental students’ overall perception of USR.

Variable	Category	Crude model					Adjusted model				
		β	PR	95% CI		p*	β	APR	95% CI		p**
				LL	UL				LL	UL	
Age group	< 22 years	-0.71	0.49	0.35	0.69	< 0.001*	-0.38	0.68	0.42	1.11	0.120
	≥ 22 years		*Ref.*					*Ref.*			
Sex	Female	-0.07	0.93	0.66	1.31	0.687	-0.17	0.84	0.61	1.18	0.319
	Male		*Ref.*					*Ref.*			
Marital status	Single	0.02	1.02	0.48	2.16	0.965	-0.06	0.94	0.44	2.01	0.880
	Married or cohabiting		*Ref.*					*Ref.*			
Type of university	Public	0.75	2.12	1.52	2.96	< 0.001*	0.92	2.51	1.67	3.78	< 0.001**
	Private		*Ref.*					*Ref.*			
Academic year	1st year	-1.18	0.31	0.17	0.55	< 0.001*	-0.54	0.58	0.29	1.18	0.136
	2nd year	-1.30	0.27	0.15	0.48	< 0.001*	-0.53	0.59	0.27	1.27	0.178
	3rd year	-0.86	0.42	0.26	0.69	0.001*	-0.08	0.92	0.52	1.64	0.784
	4th year	-0.87	0.42	0.23	0.77	0.005*	0.31	1.36	0.63	2.95	0.436
	5th year	-0.55	0.58	0.36	0.92	0.020*	0.20	1.23	0.73	2.07	0.441
	6th year		*Ref.*					*Ref.*			
Awareness of taking subjects related to USR	Yes	-0.25	0.78	0.56	1.08	0.130	-0.32	0.73	0.51	1.04	0.079
	No		*Ref.*					*Ref.*			
Model constant							-1.70	0.18	0.08	0.44	< 0.001

*Simple Poisson regression model (*p < 0.05, significant association). **Adjusted multiple regression model (**p < 0.05, significant association); PR: Prevalence Ratio; APR: Adjusted Prevalence Ratio under Poisson regression model with robust variance; β: Coefficient of determination; 95% CI: 95% Confidence Interval; LL: Lower Limit; UL: Upper Limit. The adjustment variables were age group, sex and marital status.

## Discussion

University Social Responsibility (USR) is an ethical management policy adopted by universities to address the social and environmental impacts resulting from their academic and administrative activities. It takes into account all stakeholders, including students, who play a key role in driving change and are a fundamental axis in the teaching-learning process^6,34–36^. Universities must not only include the cognitive component in professional training but also promote transversal competences of sustainability and ethics with a social component. These competences are essential for today’s democratic society^35,36^. This study aims to assess the factors associated with the perception of university social responsibility among dental students from two universities in the Peruvian capital. According to the results obtained, the null hypothesis was rejected.

The findings of the present study indicate that 16.0% of the student population had an overall poor perception of RSU. These findings differ from those reported by Mazud et al.^18^, who found that only 5% of Saudi Arabian medical students had a poor perception of USR. This discrepancy may be attributed to the fact that in the private university under consideration in this study, USR has been institutionalised through the implementation of continuous activities and the motivation of the university community to engage in USR actions through the training of leaders and teams^37–39^. However, it must be acknowledged that in the public university considered in this research, despite having an institutional licence, USR is still at an early stage of implementation, so there is a need to establish and/or reinforce strong and sustainable policies that have a positive effect on the environment and citizenship^5^. In contrast to the study by Mazud et al.^18^, whose university has institutionalised a community-based socially responsible medicine programme, which is conducted under the active supervision of a faculty member and offers various volunteer activities that allow students to participate, prepare and familiarise themselves with the target population in order to serve the community. These activities promote USR and train qualified students for community service^18,40^. As stated by Boelen^41^, Clithero et al.^42^, Roughead et al.^43^, Woolley et al.^44^, who point out that involving students in community learning sites that represent the real population ensures the acquisition of well-defined competencies for more efficient health service delivery and encourages health science students to feel the impact of their institution in the community, which enhances their perception of USR.

The results showed that 70.2% of the students had a fair perception of training in USR. This may be attributed to the fact that the Peruvian universities studied have certain strengths in cognitive issues, as they have curricula designed with external actors and also have experience in community projects, due to institutional licensing requirements. However, these do not fully encompass the Sustainable Development Goals, indicating that, although these universities are addressing these issues in a cross-cutting manner, these processes have not yet been sufficiently institutionalised^5,9^. The findings indicated that 65.6% of the students had a fair perception of the organizational management of SRU. This may be attributed to the fact that these universities have implemented exemplary measures in this domain, including fostering a positive work environment, upholding ethical standards, promoting transparency, and fostering an inclusive culture. However, these institutions have yet to implement effective environmental management practices, despite the commendable recognition and promotion of student initiatives in this domain^9^. On the other hand, it was determined that 62.1% of students had a fair perception of social participation in USR, indicating that these universities must enhance their community engagement practices. This is because they lack established and documented methodologies, and although the concept of social projection is prominent in these institutions, it is essential to recognize that the concept of USR extends beyond this, as USR prioritizes social impact. It is therefore necessary to develop this approach further in order to adapt the university’s work with society in collaboration with external actors, as this approach is still underdeveloped in Latin America^9^. These findings differ from those reported by Oriokot et al.^45^, who found that 48.1% of medical students at an East African university perceived their faculty to be performing well on social responsibility. This reflects the faculty’s efforts to achieve this goal, including community-based education, research and services, and the adoption of a competency-based medical education curriculum on this topic to better meet the needs of the community^45^.

The study revealed that dental students attending public universities were 68% more likely to have a poor perception of USR education compared to those attending private universities. This could be attributed to the fact that the term USR may be unfamiliar to students at public universities, as their curriculum, which dates back to 2006, did not emphasize this concept in their coursework. This is in line with Sebbani et al..‘s findings^13^, which showed that 35% of medical students in Morocco are not familiar with the concept of USR. Another possible explanation is that the surveyed students were taking new subjects related to USR, the contents of which are still being implemented^21^. It is important to note that the activities related to USR during the pandemic were limited, which may have affected the perception of USR training among public university students^46^. Students from private universities had already taken consolidated subjects aligned with USR in their last two curricula from the beginning of their careers, so they were familiar with the subject before, during, and after the pandemic^23^.

Furthermore, students who were aware of undertaking subjects linked to USR were 37% less likely to hold a poor perception of USR training compared to those who were not aware. These findings align with those of Coelho and Menezes^11^, who have demonstrated that students who have engaged in USR initiatives within their academic programmes develop competencies in decision-making, problem-solving, research, analysis and negotiation. This enables them to demonstrate heightened awareness, collaboration and creativity in their professional activities. This suggests that students who engage in MSR activities with a clear understanding of their purpose tend to develop a more robust understanding of the subject matter^11^. Moreover, these findings align with those of Leko et al.^15^, who demonstrated that the incorporation of a social responsibility module within the university curriculum is a significant predictor of students’ perceptions of USR. This demonstrates that the incorporation of social responsibility themes within the university curriculum fosters enhanced civic awareness among students, who cultivate a more profound comprehension of social necessities and challenges in order to effectively address these issues.

According to the results, dental students attending public universities were 2.28 times more likely to have a poor perception of organizational management in USR than those attending private universities. This may be due to the perception of public university students that organizational management is still in development, as there are currently no guidelines, plans, directives, or manuals to support USR activities^47^. This differs from a private university, where USR is already institutionalized with well-articulated management documents and curriculum. This allows for the development of activities that involve teachers, non-teaching staff, and students, who work together to improve their internal and external environment^23^.

In comparison to 6th-year students, those in their 1st and 2nd academic years were 62% and 57% less likely, respectively, to have a negative perception of organizational management in USR. This may be because first-year students perceive that university authorities recognize the importance of USR in higher education because of its educational, scientific, and cognitive nature^48^, which enables them to manage a responsible campus by adopting sustainable development practices (SDPs), unlike sixth-year students who perceived that USR management was affected during the pandemic^46,48^ because they were unable to participate in face-to-face USR activities due to mandatory social isolation. Furthermore, these sixth-year students were involved in clinical studies that emphasized care work. This finding is in line with the research conducted by Mazud et al.^18^ in Saudi Arabia, which suggests that clinical students tend to focus more on care work compared to first-year students, who have more opportunities to participate in community health promotion projects with the guidance of their teachers^18,49,50^.

The results showed that dental students in public universities were 2.31 times more likely to have a poor perception of social participation in USR compared to those in private universities. This could be attributed to the fact that students perceive that in the public university they have not yet abandoned the welfare vision towards the community and are still developing university extension and social projection activities^5^, but their USR projects are not institutionalized, and only some of them integrate the SDGs and community participation^9^. In contrast, private university students are very likely to perceive that their institution has a budget to develop USR activities because they are constantly invited to participate in social intervention projects, environmental projects, multidisciplinary research, and volunteer activities, among others, which promotes the commitment and empowerment of the educational community^51,52^ Similar results were obtained in the study of Pérez and Calvo^40^, who point out that, in a private university, medical students perceive that their institution promotes spaces for subsidized social participation since they develop intervention projects in health and the environment with a community approach, which are inserted in the context of the USR, orienting efforts towards the welfare of society.

The results indicated that dental students from public universities were 2.51 times more likely to have a poor general perception of USR compared to those from private universities. This suggests that students likely perceive that, despite being licensed and that the university law contemplates USR^53^, the public university has not yet managed to involve university stakeholders and commit the institution to develop activities in this regard, primarily due to the limited budget^40,54^. This contrasts with private universitie, which have implemented a model of USR, carrying out and disseminating activities in this regard with a higher budget^54^.

It is also important to note that cultural and contextual factors can influence students’ perceptions of USR. Universities that foster values such as respect, dignity, freedom, citizenship and social participation encourage students to participate more in USR activities. In contrast to university contexts that espouse individualistic values, the perception of USR is less positive^49^. Consequently, universities are encouraged to cultivate these values through educational policies and institutional practices, acknowledging that students’ perceptions may be shaped by the values and cultural traditions of their places of origin or experience^55^. Socio-economic background is another key factor, as students with fewer resources prioritize their basic needs over social engagement, while those with higher socio-economic status have a greater perception and willingness to engage in USR^56^. In addition, the health and environmental context can raise students’ awareness of the need for social change and sustainable practices^56,57^.

It is crucial to highlight that Peruvian legislation, through University Law 30,220^53^, has incorporated USR as an obligation in Peruvian universities. This is evidenced by the stipulation that a minimum budget of 2% must be allocated for the development of projects that promote sustainable development^58^. In addition, the university accreditation process through the National System of Evaluation, Accreditation, and Certification of Educational Quality (SINEACE) incorporates a social responsibility approach, which is reflected in the accreditation standards for academic, research, participation in social development, extension services, environmental and institutional dimensions^58^.

Despite the limited budgetary resources available to them, public universities in Peru are still required to contend with the restricted engagement and involvement of key university stakeholders in the promotion of projects that contribute to the sustainable and equitable development of the country^58^. Consequently, this research was carried out in two universities in the Peruvian capital (Lima), as a starting point for future studies. Lima is the most populous city in Peru, with an estimated population of 10,292,408 inhabitants, representing approximately 30.2% of the Peruvian population^59^. Furthermore, Lima is among the most polluted cities in Latin America^60^, and it is home to the highest concentration of dental schools in both public and private universities, representing 33.3% of all dental schools in Peru^61^. In this context, this study is significant because it prompts reflection on the current role of the university from the perspective of students. Universities are not merely obliged to educate students in a cognitive sense; they also have a responsibility to cultivate professionals who possess comprehensive skills, ethical values, and social awareness. These professionals must respond to the problems and needs of the population through sustainable solutions that facilitate the country’s development^36,46,49^. Furthermore, the findings of this study will raise the awareness of the relevant authorities to formulate strategies to improve, adapt and continue the process of implementing USR so that it can be passed on to future generations of students and contribute to the sustainable development of the nation.

It is important to note that, as of February 2024, few studies have been identified that have evaluated social responsibility in dentistry^62–64^. This indicates a need for further research in this area, with the objective of establishing strategies that incorporate USR as a management model in universities.

One of the limitations of this study is that it was not possible to make a comparison of the perception of USR among Peruvian students from rural areas, graduates, and professors. Another limitation is that the influence of cultural and contextual factors on students’ perceptions of USR was not addressed. Similarly, the cross-sectional design used did not allow for the evaluation of the dynamism and sustainability of student perception of USR over time. Finally, it should be acknowledged that, although these results cannot be extrapolated to university students from all regions of Peru, they do provide a foundation for future research aimed at promoting USR. Therefore, it is recommended that similar studies be conducted that include public and private universities from different regions of Peru and Latin America.

It should be noted that the research was conducted using the survey method, which relies on the voluntary participation of the subjects. This makes it particularly susceptible to response bias. Such bias may result in an alteration of the demographic composition of the population, which could lead to an underestimation or overestimation of students’ perceptions and, consequently, jeopardize the validity of the interpretations^65,66^. Consequently, in order to minimize the influence of response bias in this research, a number of techniques were employed to enhance the response rate (respondents were assured of anonymity, the questionnaires were presented in a clear and neutral format, and only the trained principal investigator collected the data), as well as data analysis techniques that account for non-response bias were considered^65–69^.

It is recommended that studies be carried out to assess students’ perception of MSW, considering different cultural factors, socio-economic, geographical, health and environmental contexts. It is also recommended that university authorities institutionalize USR as an integral model of organizational management through the implementation of policies that empower students in the acquisition of citizen behaviours, with the objective of achieving the sustainable development of the country^9^. Likewise, it is recommended that the necessary resources be allocated to strengthen community USR plans that include the SDGs, with particular emphasis on those related to education, health, and the environment^40,49^. It would also be advisable that academic managers involve students in USR activities such as volunteering, competitions, service-learning projects, and others from the earliest years of their professional careers, according to their formative needs^15,18^. This is to reinforce the positive perception of students towards USR, thereby fostering greater commitment to awareness, leadership, and solidarity in the service of the community^15,40,49^. Finally, it is recommended that educational interventions on USR be conducted with a longitudinal design in order to raise awareness, sensitize students on this topic, and induce them to take action.

## Conclusion

In light of the inherent limitations of this study, given the diverse contexts and challenges associated with public and private institutions, it can be concluded that more than half of the dental students exhibited a fair perception of USR. In addition, being a student at a public university was a risk factor for having a poor perception of USR. However, being aware of taking an USR-related course and being in the first years were protective factors against a poor perception of USR training and organizational management, respectively. It is recommended to involve students in USR activities that include the Sustainable Development Goals from the first years of their university education, especially in public universities.

## Electronic supplementary material

Below is the link to the electronic supplementary material.

Supplementary Material 1

## Data Availability

The datasets used and/or analysed during the current study available from the corresponding author on reasonable request.

## Abbreviations

APR: Adjusted Prevalence Ratio
CI: Confidence interval
SDGs: Sustainable Development Goals
SINEACE: National System of Evaluation, Accreditation, and Certification of Educational Quality
SPSS: Statistical Package for the Social Sciences
URSULA: Union of Latin American University Social Responsibility
USR: University Social Responsibility
WHO: World Health Organization
