# Supplementary material for: Factors associated with the perception of university social responsibility among dental students from two universities in the Peruvian capital: a multivariable regression analysis
Source: Sci Rep. 2024 Oct 7;14:23301. doi: 10.1038/s41598-024-74182-1 (PMC11458782; doi:10.1038/s41598-024-74182-1)
Supplement: Supplementary file 1 — Supplementary Material 1 [file 41598_2024_74182_MOESM1_ESM.docx]

Supplementary material: Questionnaire on University Social Responsibility

| **Perception about training in USR** | | | SD | D | N | A | SA |
| --- | --- | --- | --- | --- | --- | --- | --- |
|  |  |  | 1 | 2 | 3 | 4 | 5 |
| F1 | The university provides me with an ethical and civic education that helps me to be a socially responsible person. | |  |  |  |  |  |
| F2 | My university education is holistic and humanistic, encompassing not only professional and specialised disciplines but also broader social and cultural considerations. | |  |  |  |  |  |
| F3 | The curriculum and the formative experiences provided by the university enable me to develop proposals to address social issues. | |  |  |  |  |  |
| F4 | My university studies allow me to develop my own opinion about reality and socially relevant issues. | |  |  |  |  |  |
| F5 | The university motivates me to put myself in the place of others and to react against social and economic injustices in my social context. | |  |  |  |  |  |
| F6 | My training allows me to be an active citizen in defence of the environment and informed about the risks and ecological alternatives to current development. | |  |  |  |  |  |
| F7 | The various courses I have taken in my training are up to date and respond to the social needs of my environment. | |  |  |  |  |  |
| F8 | At the university I have the opportunity to interact with vulnerable groups in society - such as migrants, indigenous people, the elderly - who, beyond their poverty, live in situations of risk. | |  |  |  |  |  |
| F9 | As part of my training plan, I have the opportunity to participate in social projects outside the university. | |  |  |  |  |  |
| F10 | My professors link their teaching to current social and environmental problems. | |  |  |  |  |  |
| F11 | During my university education I have the possibility to meet specialists in social and environmental development issues. | |  |  |  |  |  |
| F12 | Among the activities demanded by the subjects in my curriculum, I have the opportunity to do applied research to solve social or environmental problems. | |  |  |  |  |  |
| F13 | I perceive that the assessed work and assignments have meaning for my education beyond the grade. | |  |  |  |  |  |
| F14 | The work and assignments I have done in the different courses also contribute to society, beyond my grade. | |  |  |  |  |  |
| **Perception about organizational management in USR** | | | SD | D | N | A | SA |
|  |  |  | 1 | 2 | 3 | 4 | 5 |
| G1 | | I perceive that there is a good working atmosphere at the university. |  |  |  |  |  |
| G2 | | There is respect and collaboration between professors and students at the university. |  |  |  |  |  |
| G3 | | There is respect and collaboration between administrative staff and students at the university. |  |  |  |  |  |
| G4 | | I do not perceive discrimination based on gender, race, socio-economic level or political or sexual orientation. |  |  |  |  |  |
| G5 | | I perceive that there is gender equality in access to management positions. |  |  |  |  |  |
| G6 | | I feel listened to as a citizen, I can participate in institutional life. |  |  |  |  |  |
| G7 | | The university is organised to receive students with special needs. |  |  |  |  |  |
| G8 | | The university takes measures to protect the environment on campus. |  |  |  |  |  |
| G9 | | I have acquired ecological habits since I have been at the university. |  |  |  |  |  |
| G10 | | I perceive that the university staff receives training and directives for the care of the environment on campus. |  |  |  |  |  |
| G11 | | I perceive that the processes for electing authorities are transparent and democratic. |  |  |  |  |  |
| G12 | | I perceive that the authorities take major decisions in a democratic and consensual manner. |  |  |  |  |  |
| G13 | | I perceive coherence between the principles declared by the university and what is practised on campus. |  |  |  |  |  |
| G14 | | Students are concerned about and actively participate in university life. |  |  |  |  |  |
| G15 | | Students have adequate participation in the governing institutions of the university. |  |  |  |  |  |
| G16 | | Freedom of expression and participation reigns at the university for all teaching, non-teaching and student staff. |  |  |  |  |  |
| G17 | | I am informed in a transparent manner about everything that concerns and affects me at the university. |  |  |  |  |  |
| G18 | | The university invites us to maintain good relations with other universities with which it competes. |  |  |  |  |  |
| **Perception about social participation in USR** | | | SD | D | N | A | SA |
|  |  |  | 1 | 2 | 3 | 4 | 5 |
| P1 | | My university is concerned about social problems and wants students to be agents of development. |  |  |  |  |  |
| P2 | | I perceive that my university maintains close contact with key actors in social development (state, non-governmental organisations, international organisations, companies). |  |  |  |  |  |
| P3 | | The university provides its students with opportunities to interact with various social groups such as government agencies, non-governmental organisations, international organisations, businesses, among others. |  |  |  |  |  |
| P4 | | Many forums and activities are organised at my university in relation to development, social and environmental problems. |  |  |  |  |  |
| P5 | | There is an explicit policy at the university not to segregate access to academic training for marginalised groups (indigenous population, racial minorities, low-income students, etc.) through scholarships or other means. |  |  |  |  |  |
| P6 | | At my university there are volunteering initiatives and the university encourages us to participate in them. |  |  |  |  |  |
| P7 | | In the course of my studies, I have seen that assistentialism and social development are closely related. |  |  |  |  |  |
| P8 | | Since I have been at university, I have been able to take part in groups and/or networks with social or environmental aims organised or promoted by my university. |  |  |  |  |  |
| P9 | | The students who graduate from my university have received an education that promotes their social and environmental sensitivity. |  |  |  |  |  |
| P10 | | In the course of my student life, I have been able to learn a lot about the national reality and the social problems of my country. |  |  |  |  |  |
| P11 | | In the outreach programmes and/or projects that my university carries out, the beneficiary populations are also the protagonists of development, and we students are partners in helping them in their own development. |  |  |  |  |  |
| P12 | | The outreach programmes and/or projects carried out by my university promote the integration of different disciplines as a way of tackling complex problems. |  |  |  |  |  |
| P13 | | The outreach programmes and/or projects carried out by my university generate changes or improvements in the reality of the beneficiaries, for example, by incorporating new solutions and increasing their capacities. |  |  |  |  |  |

SD: Strongly disagree, D: Disagree, N: Neutral, A: Agree, and SA: Strongly agree
